# Unravelling associations of personality traits, emotion regulation strategies, coping styles, and psychopathology with suicide risk in university students: a network perspective

**DOI:** 10.1186/s12888-025-07436-5

**Published:** 2025-10-06

**Authors:** Błażej Misiak, Dorota Frydecka, Monika Szewczuk-Bogusławska

**Affiliations:** Department of Psychiatry, Wroclaw Medical University, Wroclaw, Poland

**Keywords:** Suicidality, Stress, Coping, Personality, Emotion regulation

## Abstract

**Background:**

University students often face with adjustments to novel social contexts. This process, especially in light of maladaptive personality traits, coping styles, and emotion regulation strategies might lead to the emergence or worsening of mental disorders. Consequently, university students are likely to develop suicide-related outcomes. Therefore, in the present study, we aimed to assess the association of personality traits, emotion regulation strategies, coping styles, and psychopathology with suicide risk in university students using a network analysis.

**Methods:**

A total of 1653 university students (aged 21.6 ± 3.0 years, 71.8% females) were enrolled and assessed using self-reports administered in the internet-based survey. The analysis of a partial correlation network was carried out.

**Results:**

There were significant and positive edges of the node representing suicide risk with almost all psychopathological symptoms (except for psychotic-like experiences), avoidance coping, impulsiveness, and venturesomeness. Also, there were significant and negative connections of the node for suicide risk with problem-focused coping, cognitive reappraisal, and empathy. Among all significant connections with suicide risk, the strongest one was found for dissociation symptoms. Edge weight for this connection was significantly higher compared to all other connections with suicide risk. However, the greatest bridge expected influence was obtained from avoidance coping. It was significantly higher compared to bridge bridge expected influence of all other nodes in the network.

**Conclusions:**

Findings for the present study imply that targeting dissociation symptoms might be of importance to reduce suicide risk in university students. However, therapeutic interventions should also target on reducing avoidance coping and improving other adaptive coping strategies in order to reduce the consequences of maladaptive psychological mechanisms.

**Supplementary Information:**

The online version contains supplementary material available at 10.1186/s12888-025-07436-5.

## Introduction

Transition from adolescence to adulthood serves as the critical period that is often accompanied by the worsening of mental health [1]. This might be of importance for university students who face with challenges associated with adjustment to novel social environments. A recent systematic review estimated the pooled prevalence of depression in university students at 25% [2]. This systematic review also identified a number of risk factors related to depression in this population, including a current mental health problem, negative rumination, parent separation, a history of sexual harassment, and the presence of parental depression. There is evidence that poor mental health in university students is associated with lower program completion rates, worse educational performance, and a lower likelihood of graduation [3]. Mental health crisis in university students might also be related to an increased risk of suicide. Although it has been shown that suicide rates are lower among university students compared to the general population, rates tend to increase over time [4, 5]. Evidence from a recent meta-analysis shows that even 14% of university students might present with any suicide-related outcomes, including suicidal ideation, thoughts, and behaviors as well as completed suicides [2].

Suicide risk is increasingly understood as a multifactorial outcome, emerging from the dynamic interplay of enduring personality traits, cognitive-affective regulation, and psychopathological symptoms. Contemporary models, such as the Integrated Motivational–Volitional (IMV) model [6] and the Interpersonal Theory of Suicide [7, 8] highlight how individual differences (e.g., impulsiveness, emotional dysregulation), maladaptive coping responses, and emerging mental health symptoms can converge to elevate suicide risk. However, most studies tend to isolate these components, failing to capture their co-occurrence and mutual reinforcement in real-world populations.

Contextual factors might play important roles in the occurrence of suicide-related outcomes among university students. According to the interpersonal theory suicide developed by Joiner et al., suicide risk arises from the interplay of thwarted belongingness, perceived burdensomeness, and acquired capability [7, 8]. Perceived burdensomeness and thwarted belongingness may result from psychiatric symptomatology, e.g., depressive symptoms might contribute the feelings of being a burden and hopelessness. In turn, anxiety symptoms, dissociation, and avoidance coping may lead to both interpersonal disconnection and emotional disengagement, potentially increasing suicide risk.

In general, there is evidence that suicide-related outcomes occur as the consequence of underlying psychopathology which is perceived as the strongest predictor of suicide [9]. Indeed, various domains of psychopathology have been associated with suicide-related outcomes, including symptoms of depression [10], anxiety [11], psychosis [12], and insomnia [13]. The symptoms listed constitute core diagnostic criteria for mental disorders associated with an increased risk of suicide. Indeed, data from psychological autopsy studies revealed 40 risk factors of suicide, among them the strongest relationship was found between suicide and clinical characteristics. The diagnosis of any mental disorder followed by a history of self-harm, psychiatric treatment, a diagnosis of depression, borderline personality disorder (BPD) and schizophrenia spectrum disorders had the highest effect size within clinical domains [14]. It has also been observed that suicide-related outcomes might be associated with dissociative symptoms [15]. This domain of psychopathology often manifests in detachment experiences that are associated with decreased fear and pain sensitivity [16]. According to the DSM-5 classification, dissociation symptomatology is a criterion of BPD being highly prevalent in this clinical population [17].

Given that BPD is known as an important risk factor for suicide and in the light of the fact that a diagnosis of any personality disorder has been associated with a 20-fold higher risk of suicide compared to individuals without a psychiatric diagnosis, the contribution of specific BPD traits and those of other personality disorders in understanding the antecedents of suicide requires further attention [18]. The complexity of pathways leading to suicide-related outcomes is also associated with the effects of trait-dependent mechanisms related to personality characteristics. A recent meta-analysis revealed that higher neuroticism, higher levels of openness to experience and agreeableness as well as lower levels of extraversion and conscientiousness are associated with suicidal behaviors [19]. Some studies of suicide-related outcomes have also focused on impulsiveness that can be characterized as a multidimensional construct giving rise to behaviors that manifest in poor self-regulation, impaired planning, the occurrence of responses before thinking about potential consequences, risky behaviors, poor inhibition of responses, and the dependence on immediate rewards [20]. Some authors also posit the presence of a related construct, known as venturesomeness. As opposed to impulsiveness, this construct refers to engagement in risky behaviors having a full awareness of potential consequences [21]. There is evidence, supported by a meta-analysis, that impulsiveness, especially its affective facet, is associated with suicide-related outcomes [22]. While impulsiveness is often considered a suicide risk factor due to its role in disinhibited behaviors and poor emotional regulation [22], venturesomeness reflects a more calculated form of risk-taking, possibly relevant for premeditated suicidal behavior. Furthermore, data derived from studies on individuals who died by suicide showed that high impulsivity may predispose individuals to a greater likelihood of fatal outcomes related to suicidal behaviours [23]. Findings from other studies show that suicide attempters (SA) score higher on measures of risk-taking in comparison with healthy controls. In particular, suicide attempters have been found to report higher levels not only of thrill and sensation seeking but also of impulsive behaviour. Notably, the tendency toward risk-taking behaviours remains up to 12 weeks after suicide attempt in adult individuals placing the trait among persistent risk-factors of further suicide attempts [24, 25]. However, none of previous studies has dissected potential differences between impulsiveness and venturesomeness in shaping suicide-related outcomes. It has been shown that anticipation of suicide-related consequences might play important roles in shaping an individual decision-making capacity [26]. There is also some evidence that several suicides follow a predefined plan and thus cannot be attributed to impulsive decision-making [27]. In this regard, it might be important to differentiate the association of impulsiveness and venturesomeness that differ with respect to the presence of awareness of potential consequences. Both personality dimensions might also interact with empathy. By definition, empathy is a fairly stable personality trait [28] that enables to build social bonds, to experience the feelings of other individuals, and to accept others’ emotions, while being aware of own personal boundaries [29]. There is a paucity of studies examining the relationship between empathy and suicide risk. Since empathy is a component of emotional intelligence, it is important to note that the overall level of emotional intelligence and its specific components, including emotional awareness, emotion management, self-motivation, empathy, and relationship control, have been found significantly lower in individuals who attempted suicide [30]. Furthermore, impaired recognition of others’ emotions has been found to predispose to suicidal behaviors [31]. It has also been reported that individuals with a history of suicide attempts might be less likely to integrate emotions of other people into decision-making processes [32]. Klonsky and May [33] also suggested that a suicide attempt occurs when individual physical or emotional pain is greater than individual social connectedness.

Personality traits are further related to the way individuals process emotional states. Emotion regulation is an umbrella term for awareness, acceptance, and understanding of individual emotions together with abilities and strategies to control impulsive responses [34]. Cognitive reappraisal and expressive suppression serve as the most frequently used strategies approached to regulate emotions [35]. Cognitive reappraisal occurs at early stages of emotional processing and leads to changes of experienced emotions through their reinterpretation. In turn, expressive suppression appears at late stages of emotional processing leading to suppression of experienced emotions and manifesting, e.g., in decreased facial expression [36, 37]. Studies investigating the association between emotion regulation and various suicide-related outcomes have not provided consistent results. For instance, Forkmann et al. [38] found that expressive suppression, but not cognitive reappraisal, is associated with increased suicide ideation in an inpatient sample. In turn, another study, based on a daily diary approach, revealed that expressive suppression and cognitive reappraisal might be effective strategies among individuals with suicidal ideation [39]. Moreover, a recent systematic review revealed that emotion dysregulation is not predictive of suicidal ideation and attempts after accounting for sociodemographic characteristics, psychopathological symptoms, and a history of traumatic experiences [40].

Suicide-related outcomes often occur in the context of stressful life events. In light of stressful life events, individuals approach a variety of behaviors, commonly referred to as coping styles, that aim to manage new situations. Traditionally, coping styles can be divided into three distinct subtypes, i.e., problem-focused, emotion-focused, and avoidance coping [41–44]. There is some evidence that lower odds of using active or problem-focused coping and/or higher odds of using avoidance coping might be associated with suicidal ideation and behaviors [45–47]. Importantly, there are important conceptual differences between coping and emotion regulation [48]. For instance, emotion regulation refers to both controlled and automatic processes while coping is limited to controlled and volitional processes. Moreover, coping covers strategies employed in response to stress while emotion regulation refers to a wider context of exposures.

However, it is important to note that none of single risk factors has been found to accurately predict suicide [49]. Moreover, psychopathological symptoms show dynamic trajectories, especially in young people, and are not specific to any psychiatric diagnosis [50–52]. Consequently, dimensional approaches to psychopathology and assessment of multiple risk factors are warranted [49]. Taken together, it needs to be pointed out that discussed psychological mechanisms are usually investigated as separate constructs or predefined models of causality. However, recent advances in research of clinical psychopathology have provided a useful approach to analyze complex datasets, i.e., a network analysis. It allows to analyze multiple variables in their full spectrum of potential associations without imposing a specific model of causality. Considering existing gaps in the field, the present study aimed to investigate the association of personality traits (i.e., impulsivity, venturesomeness, and empathy), emotion regulation strategies, and coping styles with suicide risk in university students. By adopting a network analytic framework, we examined how these domains co-occur and contribute to suicide risk in a complex system, rather than testing isolated predictors.

## Material and methods

### Participants

The study included students of universities in two academic cities in Poland, i.e., Wroclaw (about 108,000 university students) and Opole (about 15,800 university students). There were two specific inclusion criteria, i.e., age ≥ 18 years and the student status at public or non-public universities in Wroclaw or Opole. Participants were enrolled during the ongoing campaign “*Talk about yourself*” that aims to open the academic society towards concerns related to mental health of university students and inform about possibilities of approaching mental health care in case of experienced crisis. The campaign was initiated in October, 2023. Participants from the sample analyzed in this study were recruited between October, 2023 and January, 2024.

Students were informed about the campaign through the websites of universities, emails, social media, flyers, radio and television interviews with mental health professionals, and printed materials disseminated in the academic society. Also, they were invited to participate in the anonymous internet-based survey. The protocol of this study was approved by the Bioethics Committee at Wroclaw Medical University, Wroclaw, Poland (approval number: 197/2023N) and all participants were consented.

### Measures

#### Psychopathological symptoms

Psychopathological symptoms assessed in the present study covered depressive and anxiety symptoms, psychotic-like experiences, dissociation symptoms, and insomnia.

To assess depressive symptoms, the Patient Health Questionnaire-8 (PHQ-8) was implemented [53]. The PHQ-8 has been developed to record the presence of depressive symptoms, e.g., “little interest or pleasure in doing things” and “feeling down, depressed, or hopeless”, over the period of preceding 2 weeks. The items are based on a four-point scale (responses range from 0 – “not at all” to 3 – “nearly every day”). The total PHQ-8 score ranges between 0 and 24. Higher scores suggest a greater severity of depressive symptoms. In the present study, the Cronbach’s alpha for the PHQ-8 was 0.837.

To measure anxiety symptoms, the Generalized Anxiety Disorder-7 (GAD-7) was used [54]. The GAD-7 refers to the presence of anxiety symptoms, e.g., “feeling nervous, anxious, or on edge” and “not being able to stop or control worrying”, over the period of preceding 2 weeks. The items are based on a four-point scale (responses range from 0 – “not at all” to 3 – “nearly every day”). The total GAD-7 score ranges between 0 and 21. Higher scores are suggestive of a greater level of anxiety symptoms. In the present study, the Cronbach’s alpha for the GAD-7 was 0.917.

To record psychotic-like experiences, the Prodromal Questionnaire-16 (PQ-16) was administered [55]. It was developed as a screening tool for the presence of the clinical high risk of psychosis. It consists of two subscales. The first one records the occurrence of psychotic-like experiences, e.g., “I have heard things other people can't hear like voices of people whispering or talking” and “I often feel that others have it in for me”, using true-or-false responses (rated as 1 or 0) while the second one refers to associated distress. In this study, we did not include the second subscale. Two items refer to the presence of depressive and anxiety symptoms (item 1: “I feel uninterested in things I used to enjoy” and item 7: “I get extremely anxious when meeting people for the first time”). To avoid the overlap of these items with those from the PHQ-8 and GAD-7, they were not included in calculating the total score. Therefore, the total score on this questionnaire ranged between 0 and 14. Higher scores indicate a greater level of psychotic-like experiences. In the present study, the Cronbach’s alpha of this 14-item questionnaire was 0.781.

To assess dissociation symptoms, the revised version of Dissociation Experiences Scale (DES) – Taxon (DES-T) was used [56]. It consists of eight items from the original DES that were found to be the most predictive for the occurrence of dissociative disorders [57]. To improve clinical utility of the DES, its revised version has been developed. This version includes a novel rating system (from 0 – “never” to 7 – “once a day or more”), based on which respondents are asked to rate the frequency of dissociative symptoms, e.g., "some people have the experience of finding themselves in a place and they have no idea how they got there” and “Some people sometimes have the experience of feeling as though they are standing next to themselves or watching themselves do something and they actually see themselves as if they were looking at another person” [56]. One item of the revised DES-T records the presence of auditory hallucinations (“some people sometimes find that they hear voices inside their head which tell them to do things or comment on things that they are doing”). To avoid the overlap of dissociation symptoms with psychotic-like experiences, this item was not included in calculating the total DES-T score. In the present study, the total DES-T score ranged between 0 and 56. The Cronbach’s alpha for the 7-item version of DES-T was 0.821 in our sample.

The Insomnia Severity Index (ISI) was used to record the occurrence of insomnia [58]. It is a seven-item measure of insomnia for the period of preceding two weeks. Three items record the type of insomnia problem (“difficulty falling asleep”, “difficulty staying asleep”, and “problems waking up too early”). Other items assess individual satisfaction with the current sleep pattern, noticeability of sleep problem to others, worry (distress) about the current sleep problem, and interference of the sleep problem with daily functioning. All items are rated on a five-point scale. The total ISI score ranges between 0 and 28. Higher scores suggest a greater level of insomnia. The Cronbach’s alpha of the ISI was 0.852 in the present study.

#### Suicide risk

The risk of suicide was assessed using the suicidality section of the Mini-International Neuropsychiatric Interview (M.I.N.I.) [59]. It is based on six questions with yes-or-no responses. Altogether, five questions capture the preceding month: “did you think that you would be better off dead or wish you were dead?” (score: 1 point), “did you want to harm yourself or to hurt or injure yourself?” (score: 2 points), “did you think about suicide?” (score: 6 points), “did you have a suicide plan?” (score: 10 points), and “did you attempt suicide?” (score: 10 points). One question refers to a lifetime history of suicide attempt (“in your lifetime, did you ever make a suicide attempt?”, score: 4 points). The risk of suicide is represented by the sum of points for responses to all questions ranging from 0 to 33. Higher scores are considered the index of a greater suicide risk. In our study, the Cronbach’s alpha of the M.I.N.I. suicidality section was 0.720.

#### Personality traits

To measure selected personality traits, the Impulsiveness-Venturesomeness-Empathy questionnaire (IVE-7) was used [21, 60]. It includes 54 items that are rated on a yes-or-no scale. Items are grouped within three subscales measuring impulsiveness, venturesomeness, and empathy. Higher scores indicate a greater level of a specific personality facet. In the present study, The Cronbach’s alpha was as follows: 0.815 for impulsiveness, 0.815 for venturesomeness, and 0.778 for empathy.

#### Coping styles

To measure coping styles, an abbreviated version of the Coping Orientation to Problems Experienced Inventory (Brief-COPE) was used [41, 61]. It includes 28 items that are rated on a four-point scale (from 1 – “I haven’t been doing this at all” to 4 – “I’ve been doing this a lot”). Coping styles assessed using the Brief-COPE cluster into three distinct coping strategies, i.e., problem-focused coping (active coping, use of informational support, planning, and positive reframing), emotion-focused coping (venting, use of emotional support, humor, acceptance, self-blame, and religion), and avoidance coping (self-distraction, denial, substance use, and behavioral disengagement). Higher total scores indicate a greater use of specific coping styles. In the present study, the Cronbach’s alphas for problem-focused coping, emotion-focused coping, and avoidant coping were 0.824, 0.741, and 0.753, respectively.

#### Emotion regulation strategies

To record emotion regulation strategies, the Emotion Regulation Questionnaire (ERQ) was administered [62]. It is a brief scale based on ten items that measures individual capacity to regulate emotions using cognitive reappraisal (six items) and expressive suppression (four items). Each item is based on a seven-point scale ranging from 1 (strongly disagree) to 7 (strongly agree). Higher scores indicate a greater use of a specific emotion regulation strategy. In the present study, the Cronbach’s alpha for cognitive reappraisal and expressive suppression subscales was 0.875 and 0.790, respectively.

### Data analysis

There were no missing data in the sample reported in this study. To assess the relationships between multiple variables assessed in this study, a network analysis was implemented. The network structure was estimated using the EBICglasso function [63]. This approach is based on the Least Absolute Shrinkage and Selection Operator (LASSO) that employs a regularizing penalty parameter to avoid indicating weak associations. In other words, weak correlations are converted exactly to zero and not visualized in the network. The extent of regularization in LASSO is selected using the penalty parameter established by minimizing the Extended Bayesian Information Criterion (EBIC) [64]. The resulting network includes variables represented by nodes that are connected with edges. Thicker edges correspond with stronger partial correlations. Blue edges indicate positive correlations while red edges represent negative associations.

After estimating the network, the bridge expected influence was calculated for all nodes [65]. It can be defined as the sum of positive and negative edges that appear between a specific node and all other nodes that are not located in the same community. As opposed to other bridge centrality metrics, it accounts for the presence of negative edges. We did not analyze node centralities as various communities contained unequal numbers of nodes, which could bias centrality metrics, such as strength, betweenness, closeness, and expected influence. In other words, the use of these metrics would result in higher centrality ranks of nodes representing communities with several variables due to a higher number of connections. Altogether, five communities of variables were predefined, including suicide risk, psychopathology, coping styles, personality traits, and emotion regulation strategies.

Next, we calculated predictability of nodes included in the network [66]. It can be defined as the percentage of variance in a specific node explained by the nodes directly connected to it. We decided to calculate these parameters to show the extent of variability in suicide risk explained by the network.

The final step covered the analysis of network stability and accuracy [63]. The case-drop and non-parametric bootstrapping procedures with 1,000 iterations were used to assess stability of the bridge expected influence and edge weights, respectively. Next, the resulting correlation stability coefficient (CS-C) was analyzed. The CS-C shows the maximum number of cases that can be removed from the data to retain a correlation of at least 0.7 between statistics of the original network and those computed with a lower number of cases. The network was considered stable if the CS-C was higher than 0.25.

All analyses were carried out in the R software (version 4.1.3). The following packages were used: *networktools* [67], *bootnet* [63], *qgraph* [68], and *mgm* [69].

## Results

**Fig. 1:**
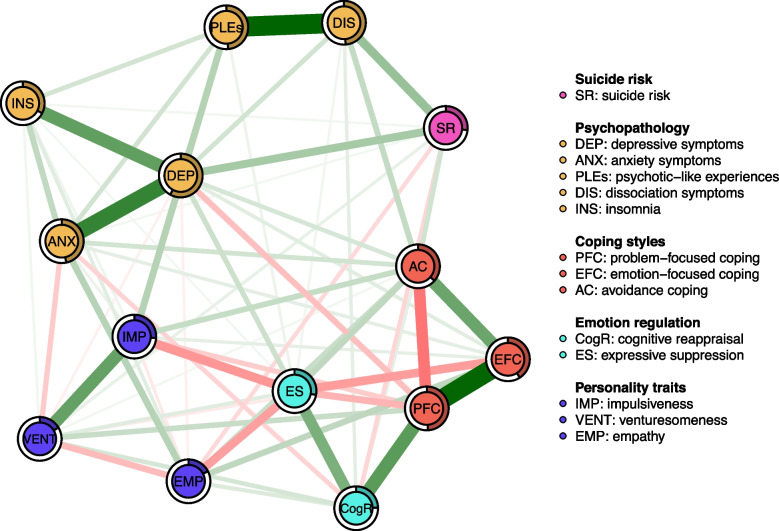
The network assessed in the present study. Filled rings around nodes show predictability

**Fig. 2:**
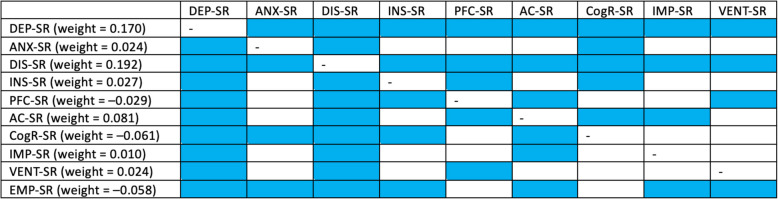
Significant connections with suicide risk in the network and their comparison. Blue boxes indicate significant differences. *Note:* AC, avoidance coping; ANX, anxiety symptoms; CogR, cognitive reappraisal; DEP, depressive symptoms; DIS, dissociation symptoms; EMP, empathy; IMP, impulsiveness; INS, insomnia; PFC, problem-focused coping; SR, suicide risk; VENT, venturesomeness

Altogether, 1653 university students were enrolled (aged 21.6 ± 3.0 years, 71.8% females, Table 1). The network analyzed in this study is shown in Fig. 1. No isolated nodes (i.e., those with a lack of connections with other nodes) were found. Out of 91 potential edges, significant correlation coefficients were found for 70 edges (76.9%, Table S1). There were significant and positive edges of the node representing suicide risk with almost all psychopathological symptoms (except for psychotic-like experiences), avoidance coping, impulsiveness, and venturesomeness (Table S1, Fig. 2). Also, there were significant and negative connections of the node for suicide risk with problem-focused coping, cognitive reappraisal, and empathy. Among all significant connections with suicide risk, the strongest one was found for dissociation symptoms (weight = 0.192, bootstrapped 95%CI: 0.121–0.227, Fig. 2). Edge weight for this connection was significantly higher compared to all other connections with suicide risk.

**Table 1:** Descriptive characteristics of the sample (*n* = 1653)

	Mean ± SD or n (%)
Age, years	21.6 ± 3.0
Gender	
Women	1187 (71.8)
Men	427 (25.8)
Other identities^*^	39 (2.4)
Lifetime history of psychiatric treatment	550 (33.3)
Lifetime history of psychological interventions	643 (38.9)
Psychiatric treatment, currently	340 (20.6)
Psychological treatment, currently	305 (18.4)
PHQ-8, depressive symptoms	12.9 ± 5.0
GAD-7, anxiety symptoms	10.7 ± 5.8
PQ-16, psychotic-like experiences^*^	4.9 ± 3.3
DES-T, dissociation symptoms^*^	6.8 ± 7.5
ISI, insomnia	9.9 ± 5.8
Brief-COPE, problem-focused coping	13.1 ± 4.9
Brief-COPE, emotion-focused coping	18.3 ± 4.9
Brief-COPE, avoidance coping	8.4 ± 3.9
ERQ, emotion regulation – cognitive reappraisal	23.9 ± 8.5
ERQ, emotion regulation – expressive suppression	15.7 ± 6.1
IVE-7, impulsiveness	6.6 ± 4.2
IVE-7, venturesomeness	7.2 ± 4.0
IVE-7, empathy	14.1 ± 3.6
Suicide risk, M.I.N.I	8.0 ± 9.6

*Brief-COPE* an abbreviated version of the Coping Orientation to Problems Experienced Inventory, *DES-T* the Dissociation Experiences Scale – Taxon, *ERQ* the Emotion Regulation Questionnaire, *GAD-7* the Generalized Anxiety Disorder-7, *ISI* the Insomnia Severity Index, *IVE-7* the Impulsiveness-Venturesomeness-Empathy 7 questionnaire, *PHQ-8* the Patient Health Questionnaire-8; the Mini-International Neuropsychiatric Interview, *PQ-16*, the Prodromal Questionnaire-16

^*^Participants who did not self-identify as men or women had the possibility to select the third option “other gender identities”

^**^The score after removing single items (see in the main text)

**Fig. 3:**
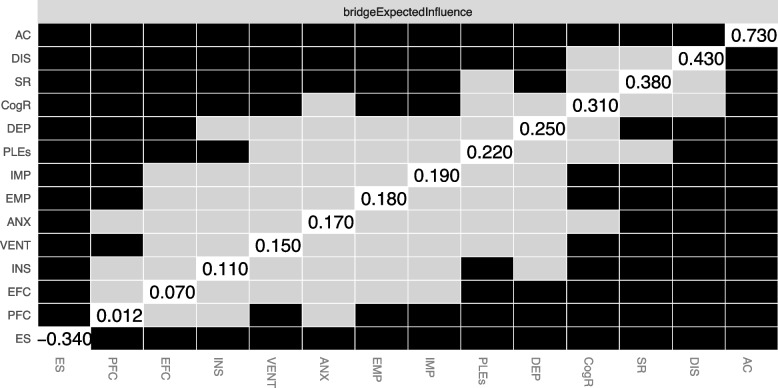
The results of bootstrapped difference tests for bridge expected influence. Black boxes refer to significant differences. *Note:* AC, avoidance coping; ANX, anxiety symptoms; CogR, cognitive reappraisal; DEP, depressive symptoms; DIS, dissociation symptoms; EMP, empathy; ES, expressive suppression; EFC, emotion-focused coping; IMP, impulsiveness; INS, insomnia; PFC, problem-focused coping; PLEs, psychotic-like experiences; SR, suicide risk; VENT, venturesomeness

The bridge expected influence is illustrated in Fig. 3. Avoidance coping not only showed a significant direct association with suicide risk but also emerged as the node with the highest bridge expected influence, suggesting it may serve as a key connector between suicide risk and other psychological domains. The bridge expected influence of avoidance coping was significantly higher compared to bridge expected influence of all other nodes in the network.

Node predictabilities are reported in Table S2. The highest predictability was obtained for depressive symptoms (0.566), while the lowest one was found for venturesomeness (0.140). In turn, suicide risk had the predictability of 0.244. The mean predictability across all nodes in the network was 0.343.

Figures S2 and S3 show the stability of bridge expected influence and edge weights, respectively. The CS-C value was 0.75 (the same value for the bridge expected influence and edge weights), indicating sufficient network stability after dropping various proportions of data.

## Discussion

### Main findings

Although the overall level of suicide risk in our non-clinical sample was low, consistent with prior prevalence studies [70], the network approach enabled us to identify meaningful associations between suicide risk and psychological variables. Findings from the present study indicate that maladaptive coping styles (i.e., decreased odds of using problem-focused coping and increased odds of using avoidance coping), lower odds of using emotion regulation through cognitive reappraisal, a higher level of impulsiveness and venturesomeness, a lower level of empathy, as well as psychopathological symptoms (except for psychotic-like experiences) are associated with a higher risk of suicide. Among these variables, the strongest association with suicide risk appeared for dissociation symptoms. It was also significantly higher compared to all other connections with suicide risk in the whole network.

The term dissociation refers to impaired integration of cognitive, emotional, motor, and behavioral processes. Recent meta-analyses revealed that dissociation symptoms, regardless of co-occurring psychiatric diagnosis, are related to increased risk of suicide attempts and non-suicidal self-injury [71]. Also, there is some evidence that dissociative symptoms better differentiate patients with a history of suicide attempt compared to depressive and post-traumatic stress disorder symptoms [72]. These observations would indicate that dissociative experiences might increase suicide capability by promoting transition within the ideation-to-action framework [73]. Specifically, dissociative experiences might lead to decreased fear and pain sensitivity thereby increasing the risk of suicide attempts among individuals with pre-existing suicidal ideation [16]. However, it is also needed to note that a recent meta-analysis demonstrated a significant association of dissociative experiences with suicidal ideation [15]. Various explanations of this association must be considered. First, dissociation itself might be a distressing experience [74] and there is evidence that non-suicidal self-injury is often acknowledged to play a role in interrupting dissociation [75]. It has been shown that individuals with conduct disorder and a lifetime history of suicide attempts who engage in non-suicidal self-injury are significantly more likely to report its anti-dissociation function [76]. Second, dissociation might lead to a lack of social connections with others and the world around thereby increasing the feelings of loneliness [74].

Importantly, the present study revealed that avoidance coping might show the greatest bridge centrality in the whole network. In other words, the strength of connections between a specific node in the network and nodes from other communities was the highest for avoidance coping. Also, this node was directly connected to a greater suicide risk. According to the network theory, the most central nodes serve as optimal targets for interventions. Their activation is supposed to activate the highest number of nodes in the network. Avoidance coping is perceived as a maladaptive strategy leading to persistence of negative thoughts, feelings, and emotions [77]. Moreover, avoidance coping has also been associated with suicide-related outcomes in general [78] and in undergraduate students specifically [47, 79, 80]. Specific mechanisms might be related to the hypothesis that avoidance reduces the ability to consider various solutions as well as to interpret and regulate emotional states leading to perceptions that suicide is the only solution to change the situation and experienced emotions [81].

Results from this study also suggest that higher levels of impulsiveness and venturesomeness as well as lower levels of empathy might be associated with a higher risk of suicide. A recent systematic review and meta-analysis, based on a multidimensional conceptualization of impulsivity, showed a significant relationship of impulsivity with suicidal ideations and behaviors [22]. Interestingly, the affective facets related to impulsivity (i.e., the tendency to act upon experiencing negative and/or positive emotions) have been shown to hold a stronger association with suicide-related outcomes in comparison with cognitive (i.e., the tendency to act without planning and consideration of consequences) and behavioral dimensions (i.e., the tendency to approach new and stimulating behaviors following sensorimotor arousal) of impulsivity. While impulsivity can be defined as thoughtless acting without regard of the consequences, venturesomeness is understood as conscious risk taking or “normal impulsivity” [82]. So far there is limited data about the association of venturesomeness with suicide-related outcomes. Previous studies have found no significant association between venturesomeness and suicidality in the population of pathological gamblers [83] and adolescents with conduct disorders [76], as well as no association with self-harming behaviors [84]. Direct associations of both impulsiveness and venturesomeness with suicide risk implies that the awareness of potential consequences differentiating both constructs may not necessarily play important roles in shaping suicide risk. These observations further support findings from a recent meta-analysis showing that affective aspects of impulsivity show stronger associations with suicide-related outcomes than its cognitive and behavioral aspects [22].

Finally, we found that the preference of cognitive reappraisal, as an emotion regulation strategy plays a role in lowering the risk of suicide. Emotion regulation, mainly the ability to regulate one’s own negative emotions, has been repeatedly shown to be associated with mental health outcomes, including the risk of suicide. A recent systematic review identified 70 studies showing that people with difficulties in emotion regulation report higher levels of suicide ideation and more suicide attempts among adolescent and adult samples as well as in clinical and general population samples [85]. One of the emotion regulation strategy is cognitive reappraisal that involves altering the emotional impact of an event by thinking about that event in a different way [62]. It has been shown that increased habitual use of cognitive reappraisal is associated with a less pronounced association between stressful events and same-day suicidal ideation [39].

### Implications

Our findings support the view that suicide-related outcomes may emerge from the interplay of interpersonal and intrapersonal vulnerabilities. For instance, dissociation and avoidance coping may contribute to acquired capability for suicide by dampening emotional and physical reactivity, whereas low empathy may reduce perceived connectedness. These patterns align with theoretical frameworks such as the Interpersonal Theory of Suicide [7] and the Ideation-to-Action model [33], emphasizing the value of integrating dimensional psychological features into suicide risk models Recognizing and targeting dissociative symptoms is one direction that needs to be considered while planning interventions for students at risk of suicide. It is needed to note that dissociative symptoms might occur across the entire spectrum of mental disorders [86]. Therefore, detecting the mechanisms of dissociation might be important for prioritizing interventions. Our observations about avoidance coping further point to the necessity to consider this target. There are studies showing that interventions focused on improving coping strategies might be effective in reducing psychiatric symptoms and suicide risk among undergraduate students and adolescents. These include therapeutic interventions, e.g., the Coping and Support Training (CAST) [87, 88], coping planning, and psychoeducation seminars [89]. Therapeutic approaches using cognitive reappraisal in order to help individuals manage emotional distress have been demonstrated to reduce emotional arousal following aversive events [90]. The value of emotional regulation has also been recognized by clinicians resulting in the development of a psychosocial intervention called the Cognitive Reappraisal Intervention for Suicide Prevention (CRISP) enhancing this emotion regulation strategy to reduce suicide risk [91]. Nevertheless, it is needed to recognize that individuals at risk of suicide often present with several vulnerabilities that are not limited to symptomatic manifestation, but also include, e.g., personality traits, traumatic experiences, unfavorable social contexts, and thus need to be prioritized. It is also needed to adopt multiple approaches that are posited within increased awareness and risk screening, proactive intervention and engagement approaches, and improved service availability [92].

### Limitations

There are some limitations of our study. First, the study was conducted in a non-clinical sample of university students that may induce a selection bias and influence generalizability of findings. At this point, it is also important to note that we did not record information about involvement of students in specific degree programmes. Second, the study included self-report measures and participants were not assessed with a detailed clinical interview using validated diagnostic instruments. Also, the use of an internet-based survey might be associated with limited data accuracy. However, this approach, in the case of anonymous character, might improve disclosure of aspects related to mental health. Moreover, a cross-sectional design of the study fails to capture within-person day-to-day fluctuations of symptoms and does not allow to infer about the causality of risk and protective factors with suicidality. Another limitation is that our network model explained 24.4% of variance in suicide risk indicating that some risk and protective factors that might be of importance were not included in the present study. Moreover, specific network connections with suicide risk were generally weak limiting their clinical relevance. Finally, our results with respect to empathy should be interpreted with caution as trans-cultural validation did not clearly identify the empathy subscale of IVE-7 [21].

## Conclusions

In summary, the network analysis revealed that personality traits (impulsivity, venturesomeness, empathy), coping mechanisms (avoidance coping), emotional regulation strategies (cognitive reappraisal), and psychopathological symptoms might be important in shaping the suicide risk. However, dissociation might show the strongest connection with suicide risk in this population. Also, taking into consideration our findings with respect to bridge centrality, it might be important to promote interventions targeting avoidance coping to improve psychological wellbeing in university students. In the future, longitudinal studies using experience sampling methods as well as detailed clinical assessments are necessary to confirm the relevance of variables assessed by the present study in shaping the risk of suicide.

## Supplementary Information

Supplementary Material 1.

## Data Availability

The datasets used and analyzed during the current study are available from the corresponding author on reasonable request.

## Abbreviations

Brief-COPE: Abbreviated version of the Coping Orientation to Problems Experienced Inventory
CSC: Correlation stability coefficient
DES-T: Dissociation Experiences Scale – Taxon
EBICglasso: Extended Bayesian Information Criterion graphical LASSO
ERQ: Emotion Regulation Questionnaire
GAD-7: Generalized Anxiety Disorder-7
ISI: Insomnia Severity Index
IVE-7: Impulsiveness-Venturesomeness-Empathy questionnaire
LASSO: Least Absolute Shrinkage and Selection Operator
M.I.N.I: Mini-International Neuropsychiatric Interview
PHQ-8: Patient Health Questionnaire-8
PQ-16: Prodromal Questionnaire-16

## References

[1] Collins A, Munoz-Solomando A. The transition from child and adolescent to adult mental health services with a focus on diagnosis progression. BJPsych Bull. 2018;42(5):188–92. 29925438 10.1192/bjb.2018.39 PMC6189989

[2] Sheldon E, Simmonds-Buckley M, Bone C, Mascarenhas T, Chan N, Wincott M, et al. Prevalence and risk factors for mental health problems in university undergraduate students: a systematic review with meta-analysis. J Affect Disord. 2021;287:282–92. 33812241 10.1016/j.jad.2021.03.054

[3] Byrd DR, McKinney KJ. Individual, interpersonal, and institutional level factors associated with the mental health of college students. J Am Coll Health. 2012;60(3):185–93. 22420695 10.1080/07448481.2011.584334

[4] Mortier P, Auerbach RP, Alonso J, Axinn WG, Cuijpers P, Ebert DD, et al. Suicidal thoughts and behaviors among college students and same-aged peers: results from the World Health Organization world mental health surveys. Soc Psychiatry Psychiatr Epidemiol. 2018;53(3):279–88. 29340781 10.1007/s00127-018-1481-6 PMC5896296

[5] Gunnell D, Caul S, Appleby L, John A, Hawton K. The incidence of suicide in University students in England and Wales 2000/2001-2016/2017: record linkage study. J Affect Disord. 2020;261:113–20. 31610312 10.1016/j.jad.2019.09.079

[6] O’Connor RC, Kirtley OJ. The integrated motivational-volitional model of suicidal behaviour. Philos Trans R Soc Lond B Biol Sci. 2018. 10.1098/rstb.2017.0268. 30012735 10.1098/rstb.2017.0268 PMC6053985

[7] Joiner TE, Van Orden KA, Witte TK, Selby EA, Ribeiro JD, Lewis R, et al. Main predictions of the interpersonal-psychological theory of suicidal behavior: empirical tests in two samples of young adults. J Abnorm Psychol. 2009;118(3):634–46. 19685959 10.1037/a0016500 PMC2846517

[8] Joiner TE. Why people die by suicide. Cambridge, MA: Harvard University Press; 2005.

[9] Turecki G, Brent DA. Suicide and suicidal behaviour. Lancet. 2016;387(10024):1227–39. 26385066 10.1016/S0140-6736(15)00234-2 PMC5319859

[10] Riera-Serra P, Navarra-Ventura G, Castro A, Gili M, Salazar-Cedillo A, Ricci-Cabello I, et al. Clinical predictors of suicidal ideation, suicide attempts and suicide death in depressive disorder: a systematic review and meta-analysis. Eur Arch Psychiatry Clin Neurosci. 2023. 10.1007/s00406-023-01716-5. 38015265 10.1007/s00406-023-01716-5 PMC11422269

[11] Bentley KH, Franklin JC, Ribeiro JD, Kleiman EM, Fox KR, Nock MK. Anxiety and its disorders as risk factors for suicidal thoughts and behaviors: a meta-analytic review. Clin Psychol Rev. 2016;43:30–46. 26688478 10.1016/j.cpr.2015.11.008 PMC4771521

[12] Yates K, Lang U, Cederlof M, Boland F, Taylor P, Cannon M, et al. Association of psychotic experiences with subsequent risk of suicidal ideation, suicide attempts, and suicide deaths: a systematic review and meta-analysis of longitudinal population studies. JAMA Psychiatr. 2019;76(2):180–9. 10.1001/jamapsychiatry.2018.3514 PMC6439738 30484818

[13] Liu RT, Steele SJ, Hamilton JL, Do QBP, Furbish K, Burke TA, et al. Sleep and suicide: a systematic review and meta-analysis of longitudinal studies. Clin Psychol Rev. 2020;81:101895. 32801085 10.1016/j.cpr.2020.101895 PMC7731893

[14] Favril L, Yu R, Uyar A, Sharpe M, Fazel S. Risk factors for suicide in adults: systematic review and meta-analysis of psychological autopsy studies. Evid Based Ment Health. 2022;25(4):148–55. 36162975 10.1136/ebmental-2022-300549 PMC9685708

[15] Pachkowski MC, Klonsky ED. The Relationship Between Dissociative Experiences and Suicide Ideation: A Meta-Analytic Review. Clin Psychol Sci Pract. 2023;31:405–16.

[16] Orbach I. Dissociation, physical pain, and suicide: a hypothesis. Suicide Life Threat Behav. 1994;24(1):68–79. 8203010

[17] Al-Shamali HF, Winkler O, Talarico F, Greenshaw AJ, Forner C, Zhang Y, et al. A systematic scoping review of dissociation in borderline personality disorder and implications for research and clinical practice: exploring the fog. Aust N Z J Psychiatry. 2022;56(10):1252–64. 35152771 10.1177/00048674221077029 PMC9511244

[18] Doyle M, While D, Mok PL, Windfuhr K, Ashcroft DM, Kontopantelis E, et al. Suicide risk in primary care patients diagnosed with a personality disorder: a nested case control study. BMC Fam Pract. 2016;17:106. 27495284 10.1186/s12875-016-0479-y PMC4974738

[19] Mota M, Ulguim HB, Jansen K, Cardoso TA, Souza LDM. Are big five personality traits associated to suicidal behaviour in adolescents? A systematic review and meta-analysis. J Affect Disord. 2024;347:115–23. 37956831 10.1016/j.jad.2023.11.002

[20] Whiteside SP, Lynam DR, Miller JD, Reynolds SK. Validation of the UPPS impulsive behaviour scale: a four-factor model of impulsivity. Eur J Pers. 2005;19(7):559–74.

[21] Caci H, Nadalet L, Bayle FJ, Robert P, Boyer P. Cross-cultural study of the Impulsiveness-Venturesomeness-Empathy Questionnaire (IVE-7). Compr Psychiatry. 2003;44(5):381–7. 14505298 10.1016/S0010-440X(03)00105-6

[22] Bruno S, Anconetani G, Rogier G, Del Casale A, Pompili M, Velotti P. Impulsivity traits and suicide related outcomes: a systematic review and meta-analysis using the UPPS model. J Affect Disord. 2023;339:571–83. 37459976 10.1016/j.jad.2023.07.086

[23] Sanz-Gomez S, Vera-Varela C, Alacreu-Crespo A, Perea-Gonzalez MI, Guija JA, Giner L. Impulsivity in fatal suicide behaviour: a systematic review and meta-analysis of psychological autopsy studies. Psychiatry Res. 2024;337:115952. 38723408 10.1016/j.psychres.2024.115952

[24] Ortin A, Lake AM, Kleinman M, Gould MS. Sensation seeking as risk factor for suicidal ideation and suicide attempts in adolescence. J Affect Disord. 2012;143(1–3):214–22. 22921521 10.1016/j.jad.2012.05.058 PMC3501599

[25] Abdoli N, Salari N, Farnia V, Khodamoradi M, Jahangiri S, Mohammadi M, et al. Risk-taking behavior among suicide attempters. J Clin Med. 2022. 10.3390/jcm11144177. 35887941 10.3390/jcm11144177 PMC9320022

[26] Huang X, Funsch KM, Park EC, Franklin JC. Anticipated consequences as the primary causes of suicidal behavior: evidence from a laboratory study. Behav Res Ther. 2020;134:103726. 32979678 10.1016/j.brat.2020.103726

[27] Smith AR, Witte TK, Teale NE, King SL, Bender TW, Joiner TE. Revisiting impulsivity in suicide: implications for civil liability of third parties. Behav Sci Law. 2008;26(6):779–97. 19039790 10.1002/bsl.848 PMC2597102

[28] Leiberg S, Anders S. The multiple facets of empathy: a survey of theory and evidence. Prog Brain Res. 2006;156:419–40. 17015094 10.1016/S0079-6123(06)56023-6

[29] Scocco P, Marietta P, Tonietto M, Dello Buono M, De Leo D. The role of psychopathology and suicidal intention in predicting suicide risk: a longitudinal study. Psychopathology. 2000;33(3):143–50. 10773773 10.1159/000029136

[30] Korkmaz S, Danaci Keles D, Kazgan A, Baykara S, Gurkan Gurok M, Feyzi Demir C, et al. Emotional intelligence and problem solving skills in individuals who attempted suicide. J Clin Neurosci. 2020;74:120–3. 32070667 10.1016/j.jocn.2020.02.023

[31] Richard-Devantoy S, Guillaume S, Olie E, Courtet P, Jollant F. Altered explicit recognition of facial disgust associated with predisposition to suicidal behavior but not depression. J Affect Disord. 2013;150(2):590–3. 23489393 10.1016/j.jad.2013.01.049

[32] Zhang K, Szanto K, Clark L, Dombrovski AY. Behavioral empathy failures and suicidal behavior. Behav Res Ther. 2019;120:103329. 30477905 10.1016/j.brat.2018.10.019 PMC6497579

[33] Klonsky ED, May AM. The Three-Step Theory (3ST): a new theory of suicide rooted in the “Ideation-to-Action” framework. Int J Cogn Ther. 2015;8:114–29.

[34] Gratz KL, Roemer L. Multidimensional assessment of emotion regulation and dysregulation: development, factor structure, and initial validation of the difficulties in emotion regulation scale. J Psychopathol Behav Assess. 2004;26(1):41–54.

[35] Yan C, Ding Q, Wang Y, Wu M, Gao T, Liu X. The effect of cognitive reappraisal and expression suppression on sadness and the recognition of sad scenes: an event-related potential study. Front Psychol. 2022;13:935007. 36211892 10.3389/fpsyg.2022.935007 PMC9537681

[36] Bebko GM, Franconeri SL, Ochsner KN, Chiao JY. Look before you regulate: differential perceptual strategies underlying expressive suppression and cognitive reappraisal. Emotion. 2011;11(4):732–42. 21707159 10.1037/a0024009

[37] Gross JJ. Antecedent- and response-focused emotion regulation: divergent consequences for experience, expression, and physiology. J Pers Soc Psychol. 1998;74(1):224–37. 9457784 10.1037//0022-3514.74.1.224

[38] Forkmann T, Scherer A, Bocker M, Pawelzik M, Gauggel S, Glaesmer H. The relation of cognitive reappraisal and expressive suppression to suicidal ideation and suicidal desire. Suicide Life Threat Behav. 2014;44(5):524–36. 24494723 10.1111/sltb.12076

[39] Franz PJ, Kleiman EM, Nock MK. Reappraisal and suppression each moderate the association between stress and suicidal ideation: preliminary evidence from a daily diary study. Cogn Ther Res. 2021;45:1120–7.

[40] Turton H, Berry K, Danaquah A, Pratt D. The relationship between emotion dysregulation and suicide ideation and behaviour: a systematic review. J Affect Disord Rep. 2021;5:100136.

[41] Carver CS. You want to measure coping but your protocol’s too long: consider the brief COPE. Int J Behav Med. 1997;4(1):92–100. 16250744 10.1207/s15327558ijbm0401_6

[42] Carver CS, Scheier MF, Weintraub JK. Assessing coping strategies: a theoretically based approach. J Pers Soc Psychol. 1989;56(2):267–83. 2926629 10.1037//0022-3514.56.2.267

[43] Carver CS, Scheier MF. Situational coping and coping dispositions in a stressful transaction. J Pers Soc Psychol. 1994;66(1):184–95. 8126648 10.1037//0022-3514.66.1.184

[44] Endler NS, Parker JD. Multidimensional assessment of coping: a critical evaluation. J Pers Soc Psychol. 1990;58(5):844–54. 2348372 10.1037//0022-3514.58.5.844

[45] Liang J, Kolves K, Lew B, de Leo D, Yuan L, Abu Talib M, et al. Coping strategies and suicidality: a cross-sectional study from China. Front Psychiatry. 2020;11:129. 32231596 10.3389/fpsyt.2020.00129 PMC7083072

[46] Benatov J, Klomek AB, Shira B, Apter A, Carli V, Wasserman C. Doing nothing is sometimes worse: comparing avoidant versus approach coping strategies with peer victimization and their association to depression and suicide ideation. J Sch Violence. 2019;19(4):456–69.

[47] Ong E, Thompson C. The importance of coping and emotion regulation in the occurrence of suicidal behavior. Psychol Rep. 2019;122(4):1192–210. 29929434 10.1177/0033294118781855 PMC6628463

[48] Compas BE, Jaser SS, Dunbar JP, Watson KH, Bettis AH, Gruhn MA, et al. Coping and emotion regulation from childhood to early adulthood: points of convergence and divergence. Aust J Psychol. 2014;66(2):71–81. 24895462 10.1111/ajpy.12043 PMC4038902

[49] Franklin JC, Ribeiro JD, Fox KR, Bentley KH, Kleiman EM, Huang X, et al. Risk factors for suicidal thoughts and behaviors: a meta-analysis of 50 years of research. Psychol Bull. 2017;143(2):187–232. 27841450 10.1037/bul0000084

[50] Caspi A, Houts RM, Ambler A, Danese A, Elliott ML, Hariri A, et al. Longitudinal assessment of mental health disorders and comorbidities across 4 decades among participants in the Dunedin birth cohort study. JAMA Netw Open. 2020;3(4):e203221. 32315069 10.1001/jamanetworkopen.2020.3221 PMC7175086

[51] Healy C, Brannigan R, Dooley N, Staines L, Keeley H, Whelan R, et al. Person-centered trajectories of psychopathology from early childhood to late adolescence. JAMA Netw Open. 2022;5(5):e229601. 35536581 10.1001/jamanetworkopen.2022.9601 PMC9092205

[52] Patel V, Saxena S, Lund C, Thornicroft G, Baingana F, Bolton P, et al. The Lancet Commission on global mental health and sustainable development. Lancet. 2018;392(10157):1553–98. 30314863 10.1016/S0140-6736(18)31612-X

[53] Kroenke K, Strine TW, Spitzer RL, Williams JB, Berry JT, Mokdad AH. The PHQ-8 as a measure of current depression in the general population. J Affect Disord. 2009;114(1–3):163–73. 18752852 10.1016/j.jad.2008.06.026

[54] Spitzer RL, Kroenke K, Williams JB, Lowe B. A brief measure for assessing generalized anxiety disorder: the GAD-7. Arch Intern Med. 2006;166(10):1092–7. 16717171 10.1001/archinte.166.10.1092

[55] Ising HK, Veling W, Loewy RL, Rietveld MW, Rietdijk J, Dragt S, et al. The validity of the 16-item version of the Prodromal Questionnaire (PQ-16) to screen for ultra high risk of developing psychosis in the general help-seeking population. Schizophr Bull. 2012;38(6):1288–96. 22516147 10.1093/schbul/sbs068 PMC3713086

[56] Pietkiewicz IJ, Hełka AM, Tomalski R. Validity and reliability of the revised Polish online and pen-and-paper versions of the Dissociative Experiences Scale (DESR-PL). Eur J Trauma Dissociation. 2019. 10.1016/j.ejtd.2019.02.003.

[57] Waller NG, Putnam FW, Carlson EB. Types of dissociation and dissociative types: a taxometric analysis of dissociative experiences. Psychol Methods. 1996;1(3):300–21.

[58] Morin CM, Belleville G, Belanger L, Ivers H. The insomnia severity index: psychometric indicators to detect insomnia cases and evaluate treatment response. Sleep. 2011;34(5):601–8. 21532953 10.1093/sleep/34.5.601 PMC3079939

[59] Sheehan DV, Lecrubier Y, Sheehan KH, Amorim P, Janavs J, Weiller E, et al. The Mini-International Neuropsychiatric Interview (M.I.N.I.): the development and validation of a structured diagnostic psychiatric interview for DSM-IV and ICD-10. J Clin Psychiatry. 1998;59 Suppl 20:22–33;quiz 4–57. 9881538

[60] Eysenck SBG, Eysenck HJ. Impulsiveness and venturesomeness: their position in a dimensional system of personality description. Psychol Rep. 1978;43(3):1247–55. 746091 10.2466/pr0.1978.43.3f.1247

[61] Jurczyński Z, Ogińska-Bulik N. Narzędzia Pomiaru Stresu i Radzenia Sobie Ze Stresem. Warsaw: Psychological Test Laboratory of the Polish Psychological Association; 2012.

[62] Gross JJ, John OP. Individual differences in two emotion regulation processes: implications for affect, relationships, and well-being. J Pers Soc Psychol. 2003;85(2):348–62. 12916575 10.1037/0022-3514.85.2.348

[63] Epskamp S, Borsboom D, Fried EI. Estimating psychological networks and their accuracy: a tutorial paper. Behav Res Methods. 2018;50(1):195–212. 28342071 10.3758/s13428-017-0862-1 PMC5809547

[64] Foygel R, Drton M. Extended Bayesian information criteria for Gaussian graphical models. Advances in Neural Information Processing Systems. 2010;1:604–12.

[65] Jones PJ, Ma R, McNally RJ. Bridge centrality: a network approach to understanding comorbidity. Multivar Behav Res. 2021;56(2):353–67. 10.1080/00273171.2019.1614898 31179765

[66] Haslbeck JMB, Waldorp LJ. How well do network models predict observations? On the importance of predictability in network models. Behav Res Methods. 2018;50(2):853–61. 28718088 10.3758/s13428-017-0910-x PMC5880858

[67] Jones PJ. networktools: Assorted Tools for Identifying Important Nodes in Networks. R package version 1.0.0. 2017. p. https://CRAN.R-project.org/package=networktools.

[68] Epskamp S, Cramer AOJ, Waldorp LJ, Schmittmann VD, Borsboom D. Qgraph: network visualizations of relationships in psychometric data. J Stat Softw. 2012;48(4):1–18.

[69] Haslbeck JMB, Waldorp LJ. MGM: estimating time-varying mixed graphical models in high-dimensional data. J Stat Softw. 2020;93(8):1–46.

[70] Mortier P, Cuijpers P, Kiekens G, Auerbach RP, Demyttenaere K, Green JG, et al. The prevalence of suicidal thoughts and behaviours among college students: a meta-analysis. Psychol Med. 2018;48(4):554–65. 28805169 10.1017/S0033291717002215

[71] Calati R, Bensassi I, Courtet P. The link between dissociation and both suicide attempts and non-suicidal self-injury: meta-analyses. Psychiatry Res. 2017;251:103–14. 28196773 10.1016/j.psychres.2017.01.035

[72] Webermann AR, Myrick AC, Taylor CL, Chasson GS, Brand BL. Dissociative, depressive, and PTSD symptom severity as correlates of nonsuicidal self-injury and suicidality in dissociative disorder patients. J Trauma Dissociation. 2016;17(1):67–80. 26211678 10.1080/15299732.2015.1067941

[73] Bayliss LT, Christensen S, Lamont-Mills A, du Plessis C. Suicide capability within the ideation-to-action framework: a systematic scoping review. PLoS ONE. 2022;17(10):e0276070. 36301944 10.1371/journal.pone.0276070 PMC9612581

[74] Cernis E, Freeman D, Ehlers A. Describing the indescribable: a qualitative study of dissociative experiences in psychosis. PLoS ONE. 2020;15(2):e0229091. 32074139 10.1371/journal.pone.0229091 PMC7029850

[75] Klonsky ED. The functions of deliberate self-injury: a review of the evidence. Clin Psychol Rev. 2007;27(2):226–39. 17014942 10.1016/j.cpr.2006.08.002

[76] Szewczuk-Boguslawska M, Kaczmarek-Fojtar M, Halicka-Maslowska J, Misiak B. Self-Injuries and Their Functions with Respect to Suicide Risk in Adolescents with Conduct Disorder: Findings from a Path Analysis. J Clin Med. 2021;10(19):4602. 10.3390/jcm10194602 PMC8509303 34640620

[77] Zuckerman M, Gagne M. The COPE revised: proposing a 5-factor model of coping strategies. J Res Pers. 2003;37(3):169–204.

[78] Rogier G, Chiorri C, Beomonte Zobel S, Muzi S, Pace CS, Cheung MW, et al. The multifaceted role of emotion regulation in suicidality: systematic reviews and meta-analytic evidence. Psychol Bull. 2024;150(1):45–81. 38376911 10.1037/bul0000415

[79] Chou WP, Yen CF, Liu TL. Predicting effects of psychological inflexibility/experiential avoidance and stress coping strategies for internet addiction, significant depression, and suicidality in college students: a prospective study. Int J Environ Res Public Health. 2018. 10.3390/ijerph15040788. 29670025 10.3390/ijerph15040788 PMC5923830

[80] Tang F, Qin P. Influence of personal social network and coping skills on risk for suicidal ideation in Chinese university students. PLoS ONE. 2015;10(3):e0121023. 25803665 10.1371/journal.pone.0121023 PMC4372485

[81] Chawla N, Ostafin B. Experiential avoidance as a functional dimensional approach to psychopathology: an empirical review. J Clin Psychol. 2007;63(9):871–90. 17674402 10.1002/jclp.20400

[82] Cottraux J, Note ID, Boutitie F, Milliery M, Genouihlac V, Yao SN, et al. Cognitive therapy versus Rogerian supportive therapy in borderline personality disorder. Two-year follow-up of a controlled pilot study. Psychother Psychosom. 2009;78(5):307–16. 10.1159/000229769 19628959

[83] Ledgerwood DM, Petry NM. Gambling and suicidality in treatment-seeking pathological gamblers. J Nerv Ment Dis. 2004;192(10):711–4. 15457117 10.1097/01.nmd.0000142021.71880.ce

[84] Halicka-Maslowska J, Szewczuk-Boguslawska M, Rymaszewska J, Adamska A, Misiak B. From emotional intelligence to self-injuries: a path analysis in adolescents with conduct disorder. Front Psychiatry. 2020;11:556278. 33488414 10.3389/fpsyt.2020.556278 PMC7819897

[85] Colmenero-Navarrete L, Garcia-Sancho E, Salguero JM. Relationship between emotion regulation and suicide ideation and attempt in adults and adolescents: a systematic review. Arch Suicide Res. 2022;26(4):1702–35. 34821201 10.1080/13811118.2021.1999872

[86] Lyssenko L, Schmahl C, Bockhacker L, Vonderlin R, Bohus M, Kleindienst N. Dissociation in psychiatric disorders: a meta-analysis of studies using the Dissociative Experiences Scale. Am J Psychiatry. 2018;175(1):37–46. 28946763 10.1176/appi.ajp.2017.17010025

[87] Keliat BA, Tololiu TA, Daulima NHC, Erawati E. The influence of the training of coping skills for stress on self-control and intensity of depression among adolescents with suicide risk. Int J Adv Nurs Stud. 2015;4(2):110–4.

[88] Thompson EA, Eggert LL, Randell BP, Pike KC. Evaluation of indicated suicide risk prevention approaches for potential high school dropouts. Am J Public Health. 2001;91(5):742–52. 11344882 10.2105/ajph.91.5.742 PMC1446664

[89] Till B, Hofhansl A, Niederkrotenthaler T. Effects of the mental health promotion seminar “Coping with stress” in the undergraduate medical curriculum of the Medical University of Vienna. BMC Med Educ. 2024;24(1):41. 38191363 10.1186/s12909-023-05019-0 PMC10773058

[90] Troy AS, Saquib S, Thal J, Ciuk DJ. The regulation of negative and positive affect in response to daily stressors. Emotion. 2019;19(5):751–63. 30148373 10.1037/emo0000486

[91] Kiosses DN, Alexopoulos GS, Hajcak G, Apfeldorf W, Duberstein PR, Putrino D, et al. Cognitive reappraisal intervention for suicide prevention (CRISP) for middle-aged and older adults hospitalized for suicidality. Am J Geriatr Psychiatry. 2018;26(4):494–503. 29395858 10.1016/j.jagp.2017.11.009 PMC5860974

[92] McKay S, Veresova M, Bailey E, Lamblin M, Robinson J. Suicide prevention for international students: a scoping review. Int J Environ Res Public Health. 2023. 10.3390/ijerph20021500. 36674253 10.3390/ijerph20021500 PMC9865713

