# Supplementary material for: Unravelling associations of personality traits, emotion regulation strategies, coping styles, and psychopathology with suicide risk in university students: a network perspective
Source: BMC Psychiatry. 2025 Oct 6;25:934. doi: 10.1186/s12888-025-07436-5 (PMC12502196; doi:10.1186/s12888-025-07436-5)

**Supplementary Appendix**

**The list of abbreviations:**

- AC, avoidance coping
- ANX, anxiety symptoms
- CogR, cognitive reappraisal
- DEP, depressive symptoms
- DIS, dissociation symptoms
- EMP, empathy
- ES, expressive suppression
- EFC, emotion-focused coping
- EMP, empathy
- IMP, impulsiveness
- INS, insomnia
- PFC, problem-focused coping
- PLEs, psychotic-like experiences
- SR, suicide risk
- VENT, venturesomeness

**Table S1.** Weights matrix.


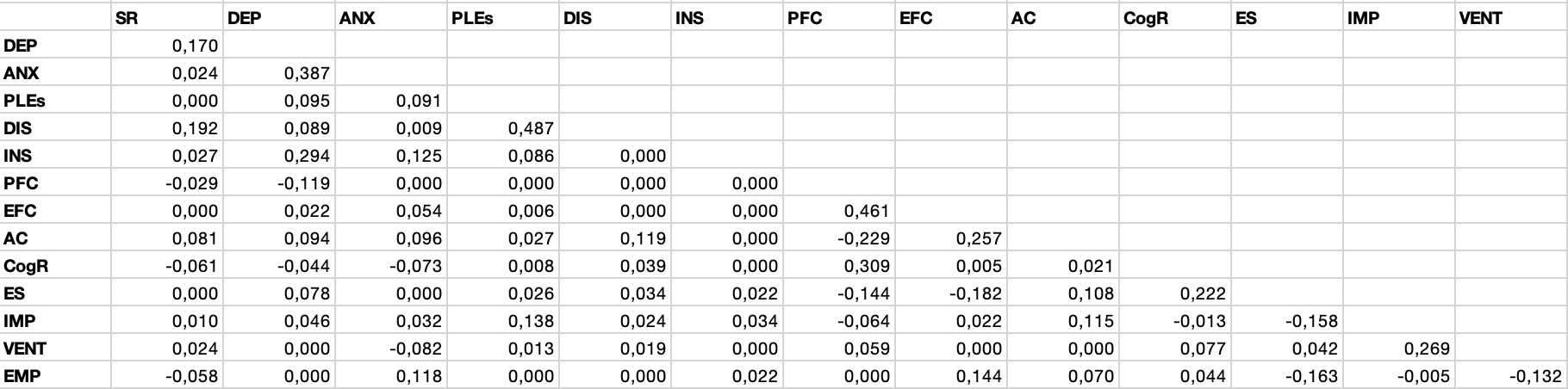


**Table S2.** Node predictabilities.

| Node | Predictability |
| --- | --- |
| SR | 0.244 |
| DEP | 0.566 |
| ANX | 0.455 |
| PLEs | 0.482 |
| DIS | 0.480 |
| INS | 0.315 |
| PFC | 0.486 |
| AC | 0.359 |
| CogR | 0.237 |
| ES | 0.278 |
| IMP | 0.249 |
| VENT | 0.140 |
| EMP | 0.167 |

**Figure S1.** The comparison of edge weights in the network. Black boxes refer to significant differences, while grey boxes mark differences that appeared to be not significant.


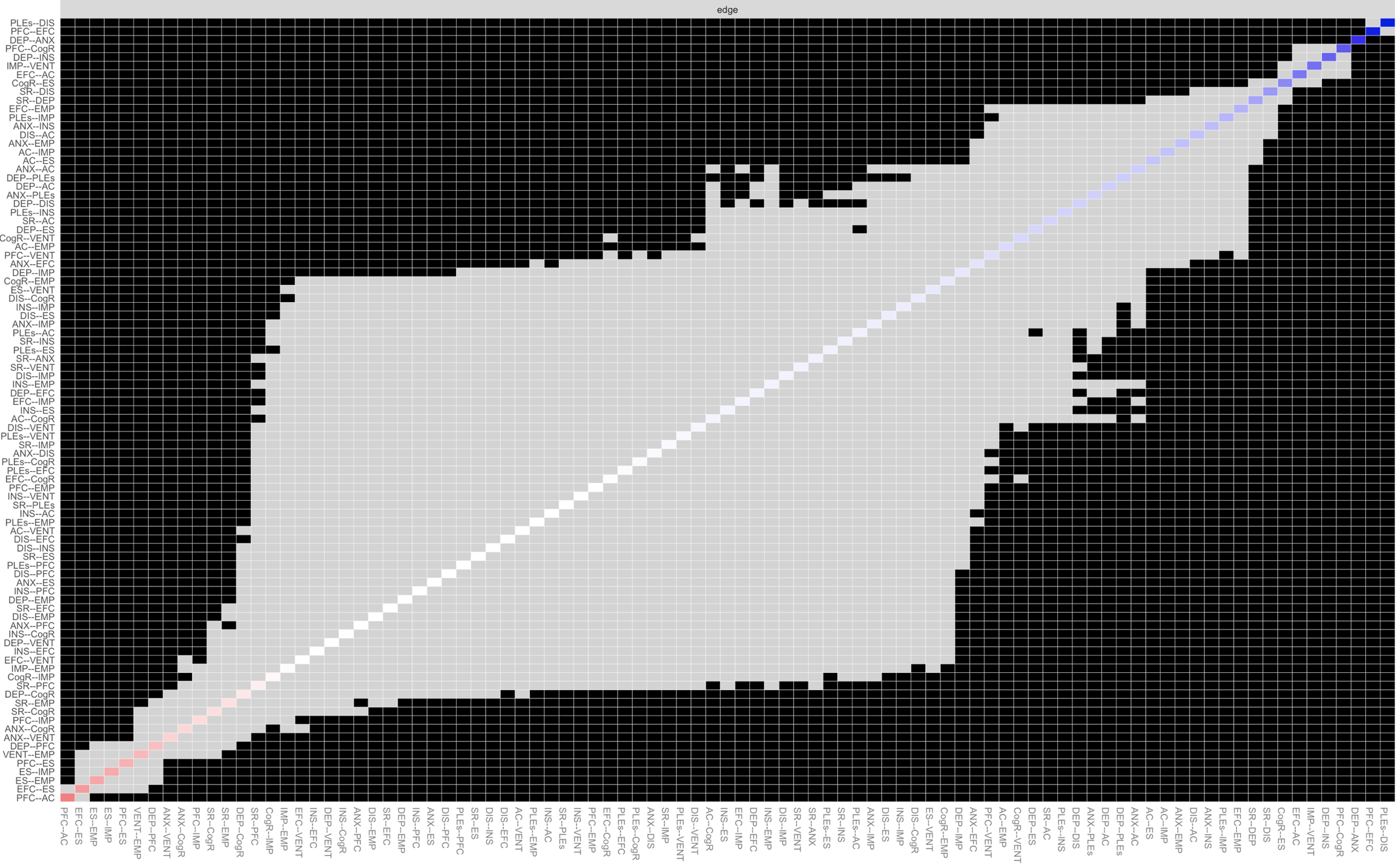


**Figure S2.** Stability of the bridge expected influence index. The red line shows changes of the centrality index after dropping various proportions of data.

**Figure 3.** Bootstrapped 95% confidence intervals of edge weights. The sample values are illustrated using a red line. The bootstrapped 95% confidence intervals are depicted within the grey area.


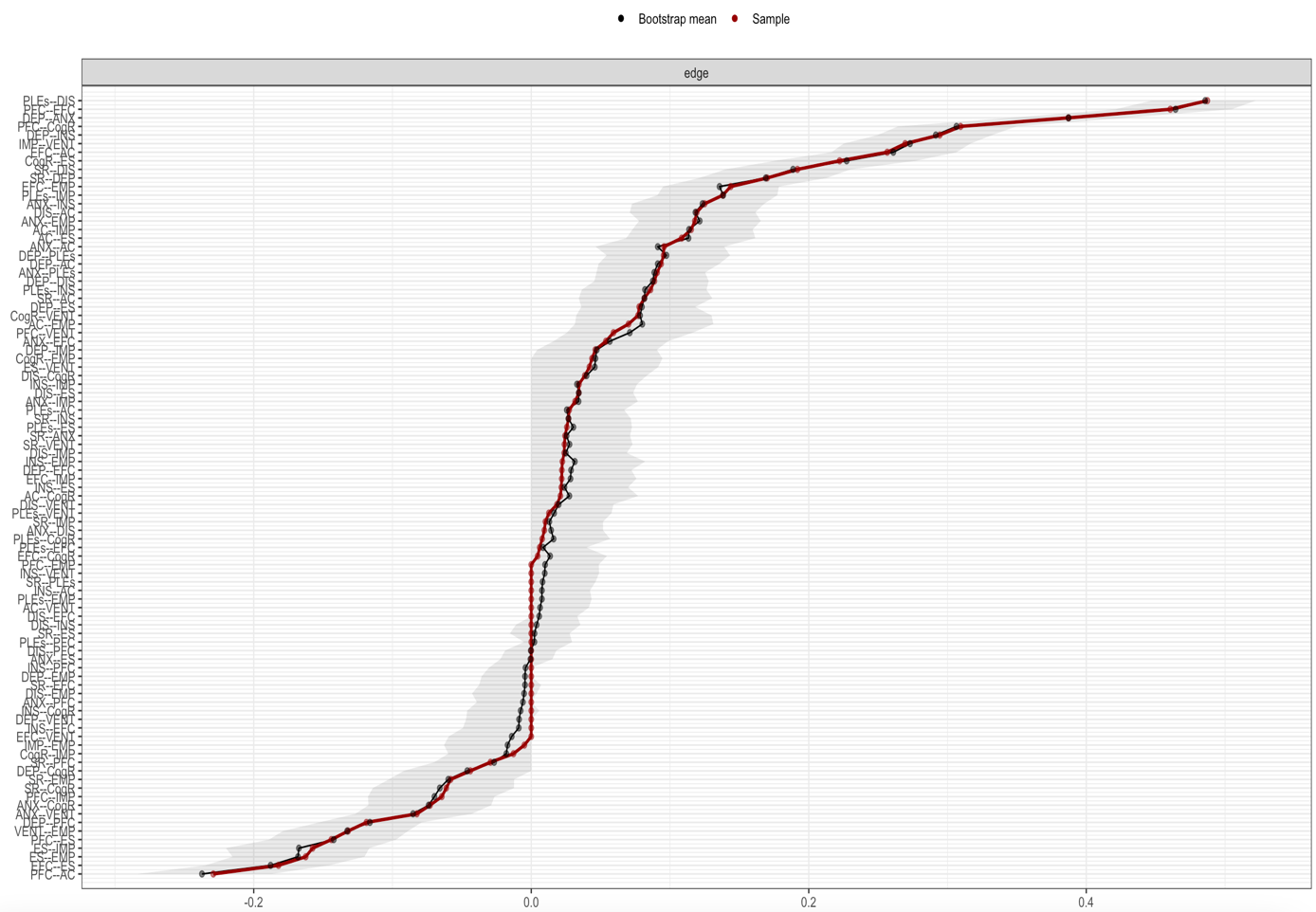

Supplement: Supplementary file 1 — Supplementary Material 1. [file 12888_2025_7436_MOESM1_ESM.docx]
